# Quantitative separation of the anisotropic magnetothermopower and planar Nernst effect by the rotation of an in-plane thermal gradient

**DOI:** 10.1038/srep40586

**Published:** 2017-01-17

**Authors:** Oliver Reimer, Daniel Meier, Michel Bovender, Lars Helmich, Jan-Oliver Dreessen, Jan Krieft, Anatoly S. Shestakov, Christian H. Back, Jan-Michael Schmalhorst, Andreas Hütten, Günter Reiss, Timo Kuschel

**Affiliations:** 1 Center for Spinelectronic Materials and Devices, Department of Physics, Bielefeld University, Universitätsstraße 25, 33615 Bielefeld, Germany; 2 Institute of Experimental and Applied Physics, University of Regensburg, Universitätsstraße 31, 93040 Regensburg, Germany; 3 Physics of Nanodevices, Zernike Institute for Advanced Materials, University of Groningen, Nijenborgh 4, 9747 AG Groningen, The Netherlands

## Abstract

A thermal gradient as the driving force for spin currents plays a key role in spin caloritronics. In this field the spin Seebeck effect (SSE) is of major interest and was investigated in terms of in-plane thermal gradients inducing perpendicular spin currents (transverse SSE) and out-of-plane thermal gradients generating parallel spin currents (longitudinal SSE). Up to now all spincaloric experiments employ a spatially fixed thermal gradient. Thus, anisotropic measurements with respect to well defined crystallographic directions were not possible. Here we introduce a new experiment that allows not only the in-plane rotation of the external magnetic field, but also the rotation of an in-plane thermal gradient controlled by optical temperature detection. As a consequence, the anisotropic magnetothermopower and the planar Nernst effect in a permalloy thin film can be measured simultaneously. Thus, the angular dependence of the magnetothermopower with respect to the magnetization direction reveals a phase shift, that allows the quantitative separation of the thermopower, the anisotropic magnetothermopower and the planar Nernst effect.

Adding the spin degree of freedom to conventional charge-based electronics opens the field of spintronics^1^,^2^ with promising advantages such as decreased electric power consumption and increased integration densities. While spinelectronics use only voltages as driving force for currents, thermal gradients and the interaction between spins and heat currents have already been shown to provide new effects. Spin caloritronics investigate these interactions and promotes the search for applications such as heat sensors or waste heat recyclers^3^,^4^, that can improve thermoelectric devices.

One of the most important and well established phenomena in spin caloritronics is the longitudinal spin Seebeck effect (LSSE)^5^,^6^,^7^,^8^,^9^,^10^,^11^, which uses typically out-of-plane thermal gradients in magnetic thin films for the generation of a spin current parallel to the thermal gradient. This pure spin current is then injected into an adjacent non-magnetic conductor with high spin-orbit coupling, e.g. Pt, which transforms the spin current into an electric voltage via the inverse spin Hall effect (ISHE). In very recent investigations, the LSSE was even detected without any Pt and ISHE by the use of the anomalous Hall effect in Au^12^ and by time-resolved magnetooptic Kerr effect in Au and Cu^13^.

Besides the application of an out-of-plane thermal gradient, effects driven by in-plane thermal gradients were also investigated. The transverse spin Seebeck effect (TSSE), the spin current generation perpendicular to an in-plane thermal gradient, was reported for metals^14^, semiconductors^15^ and insulators^16^. However, it has been noted that TSSE experiments in metals and semiconductors can be influenced by parasitic effects like the planar Nernst effect (PNE)^17^ or the anomalous Nernst effect (ANE)^18^. The first occurs in samples with magnetic anisotropy^19^, while the latter can be attributed to unintended out-of-plane temperature gradients^20^ due to heat flux into the surrounding region^21^ or through the electric contacts^22^. Recently, the influence of inhomogeneous magnetic fields was added to the list of uncertainties for TSSE experiments^23^. Despite first reports, the TSSE could not be reproduced neither for metals^19^,^20^,^21^,^22^,^23^,^24^, for semiconductors^25^ nor insulators^26^.

Closely related to the recently reported spin Hall magnetoresistance (SMR)^27^,^28^,^29^,^30^ are magnetothermopower effects that were detected in bilayers of nonmagnetic conductors/ferromagnetic insulators^26^,^31^. In the first case an in-plane electric current is driven through a conductor with high spin-orbit coupling (e.g. Pt) deposited on a magnetic insulator. The interplay of the spin Hall effect (SHE) and the ISHE induces an anisotropic electric resistance in the conductor depending on the relative orientation between the spin polarization of the normal metal and the magnetization of the magnetic insulator. Whereas the SMR uses an electric potential to inject the charge current, the so-called spin Nernst magnetothermopower is driven by an in-plane thermal gradient and can be described by the recently discovered spin Nernst effect^31^,^32^ in combination with the ISHE. Hints of these effects were already observed as side-effects in experiments using heatable electric contact tips^26^. This is another example for the use of in-plane thermal gradients in spin caloritronics. However, the in-plane thermal gradients used so far are spatially fixed.

Different techniques to apply thermal gradients include Joule heating in an external heater^9^,^14^, laser heating^8^,^33^, Peltier heating^5^,^21^,^23^, current-induced heating in the sample^34^, heating with electric contact needles^22^,^26^ and on-chip heater devices^35^. In this work we present a setup, which uses Peltier heating as a method for heating and cooling purposes to cover a larger working temperature range. As a key feature, this setup allows the in-plane rotation of a thermal gradient 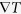 and, thus, also the angle-dependent investigation of the anisotropic magnetothermopower (AMTP) and planar Nernst effect (PNE). The use of an infrared camera optically resolves the rotation of 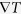.

In order to demonstrate the functionality of the setup, we will concentrate on the electric characterization of magnetothermopower effects, which can be measured under different angles of 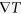. In analogy to the description of the anisotropic magnetoresistance, one can derive similar equations for magnetothermopower effects (supplementary information (SI) chapter I). The setup of our experiment and the definition of the directions of 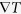, and the external magnetic field 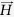 with respect to the coordinates are sketched in Fig. 1(a). When a temperature gradient 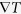 is applied, its y-component 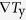 will generate a longitudinal AMTP and thus an electric field *E*_y_ in the y-direction. This longitudinal AMTP can be described by


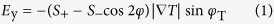


with 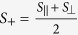, 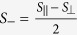 and *S*_‖_, *S*_⊥_ being the Seebeck coefficients of the thermopower parallel and perpendicular to 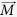, respectively. *S*_+_ originates from the ordinary, magnetic field independent thermovoltage whereas *S*_−_ describes the magnetic field dependent part of the AMTP. *φ* and *φ*_T_ are the angles of the external magnetic field and 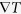, respectively, with respect to the x-axis as defined in Fig. 1(a). The transverse magnetothermopower also contributes to *E*_y_ but is driven by 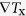 and will be denoted as the PNE, which is determined by


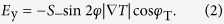


Summing up the AMTP and PNE contributions in the y-direction, we end up with


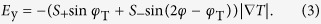


Thus, the angle *φ*_T_ of the thermal gradient acts as a phase shift for the magnetization dependent part *S*_−_ of the thermopower.

In Sec. I, the functionality of the setup is briefly explained and the measurement modes are introduced. In Sec. II, the setup is used to characterize the AMTP and the PNE in a thin Ni_80_Fe_20_ (Py) film depending on the rotation angle *φ*_T_ of 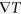. Increasing *φ*_T_ leads to a phase shift in *V*_y_ and Eqs (1) and (2) are used to split the superimposed voltage signals into the contributions of the AMTP and PNE. This enables a determination of the Seebeck coefficients parallel and perpendicular to the magnetization of the sample.

## Experimental Setup

**Figure 1:**
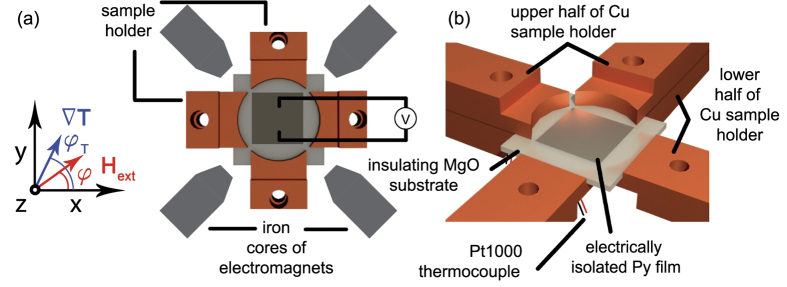
(**a**) The sample is clamped without thermal grease between four circularly shaped copper holders, which can be heated independently. Thus, the variation of different applied 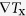 and 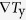 results in rotated net 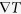. Due to its centered deposition, the Py film is electrically insulated from the sample holder. Two pairs of electromagnets rotated by ±45° with respect to the x axis supply a rotatable in-plane magnetic field based on the superposition of the fields of both magnetic axes. (**b**) Each sample holder consists of a lower and upper half to reduce unintended out-of-plane thermal gradients in the sample. The temperatures are detected via PT1000 elements attached ≈2 mm next to the sample.

The setup realizes an in-plane rotation of 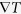 by four independently heated sample holders (Fig. 1(a)). The sample is clamped in the center of the sample holders and the application of different x and y temperature differences leads to a superpositioned net thermal gradient along *φ*_T_. Four electromagnets arranged as shown in Fig. 1(a) additionally provide a rotatable in-plane magnetic field along *φ*. All electric measurements were conducted along the y axis of a sputter deposited Py thin film (5 × 5 mm^2^, 18 nm thick) on MgO(001). To reduce parasitic effects induced by unintended out-of-plane 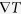, the heat is transferred into the sample using an upper and a lower half of the sample holder (Fig. 1(b)). This was already used in previous setups^22^,^26^ and could successfully reduce unintended out-of-plane thermal gradients. PT1000 elements are glued at the backsides of each sample holder to detect the temperatures of the sample holders.

**Figure 2:**
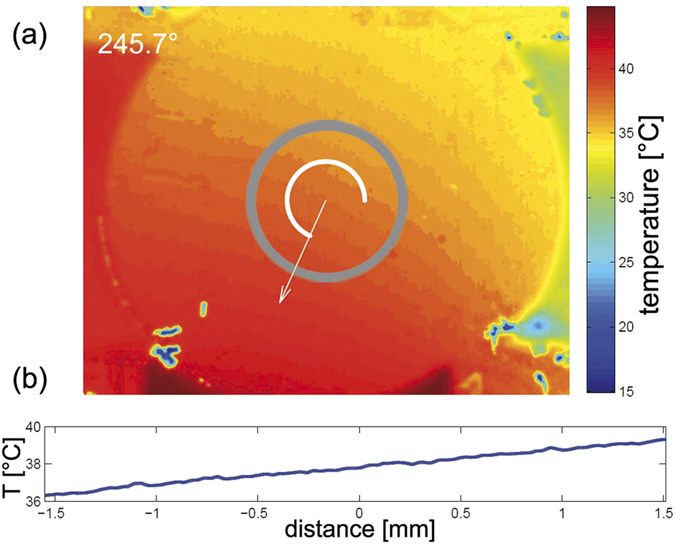
(**a**) Thermographic picture of a Cu substrate, coated with high-absorbing clustered Au particles, with applied 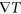 at *φ*_T_ = 240°. The gray circle represents the ROI, in which an averaged angle of 245.7° was calculated. (**b**) Temperature profile along *φ*_T_ = 245.7°.

The successful rotation of 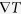 is proven by an infrared camera for MgO and Cu substrates, covered by high-absorbing Au clusters deposited under nitrogen atmosphere. The infrared measurements clearly resolve the rotation of 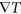 (see SI chapter II, with refs 36, 37, 38). Figure 2(a) shows a thermographic picture of a Cu substrate with 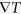 applied along *φ*_T_ = 240°. After defining a Region of Interest (ROI, gray circle) the average angle of 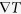 within the ROI can be calculated, symbolized by the white arc. Here, a deviation of the applied angle and the calculated angle of ≈6° is detected. Taking a relative rotation between the setup and the camera by 2° into account, a mismatch of 4° is denoted as the uncertainty of *φ*_T_.

For the quantitative analysis, *V*_y_ is averaged over five single measurements while the sample was kept at a base temperature of 308 K. When *V*_y_ is measured as a function of the external magnetic field 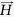, which is varied from −150 Oe up to +150 Oe (black branch of results) and back down to −150 Oe (red branch of results), the measurement mode will be denoted as *sweep measurement*. When *V*_y_ is measured in magnetic saturation as a function of *φ*, the *field rotation measurement* mode was used. Here, the magnetization was kept saturated along the direction of 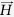 (Δ*φ* = ±3°) by using an external magnetic field of 200 Oe, which then was rotated counterclockwise in the x-y plane (Fig. 1(a)).

## Results

### Δ*T* dependence of the PNE

**Figure 3:**
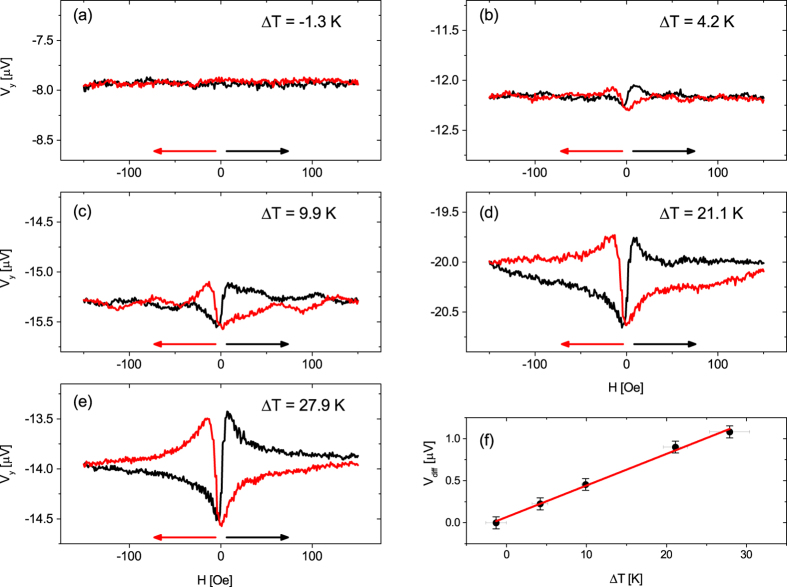
(**a**–**e**) *V*_y_ as a function of magnetic field for increasing Δ*T* and *φ* = *φ*_T_ = 0°. (**f**) *V*_diff_ = *V*_max_ − *V*_min_ was calculated and averaged for each branch of each Δ*T* and plotted as a function of Δ*T*, showing the expected linear dependence (Eq. (2)).

Figure 3 shows sweep measurements of *V*_y_ with 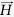 aligned along *φ* = 0°. Here, Δ*T* was increased from ≈0 K to ≈30 *K* along *φ*_T_ = 0°. Keeping 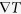 along the x direction and measuring the voltage only in the y direction excludes any AMTP contributions so that *V*_y_ in Fig. 3 only shows the PNE. In Fig. 3(a) a very low Δ*T* is applied along the x axis, which is too low to induce a detectable voltage along the y axis. Therefore, only the noise level (≈50 nV) can be recorded. Depending on Δ*T, V*_y_ shows increasing peaks in the low magnetic field regime, and saturates for 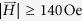 (Fig. 3(a–e)).

A similar experiment was conducted by Meier *et al*.^22^, which is in good agreement with the data shown in Fig. 3. Slight deviations of the signal shape can be attributed to different magnetic anisotropies for different samples and small parasitic magnetic fields of the electromagnet due to the interaction of both magnetic axes (see SI chapter III). Starting with the increase of the magnetic field from −150 Oe to +150 Oe, for low negative field values the voltage of the PNE measurement (e.g. Fig. 3(e), black branch) first drops to a minimum voltage, lower than the saturation voltage, before it rises to a maximum value above the saturation voltage. Only then it decreases and saturates again. While decreasing the magnetic field after its maximum (red branch), again first the development of a minimum and then of a maximum is observed, before the voltage approaches the initial saturation value.

For verifying the temperature dependence of the PNE, the peak-to-peak height in the low magnetic field regime is chosen as an indication of the PNE strength. The peak-to-peak height is quantified by *V*_diff_, calculating the voltage difference between the maximum and minimum voltage for each branch and averaging them. Figure 3(f) shows *V*_diff_ vs. Δ*T*. This correlation can be fitted linearly and therefore confirms the proportionality to Δ*T*, as can be seen in Eq. (2).

### 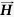 angular dependence of the PNE

**Figure 4:**
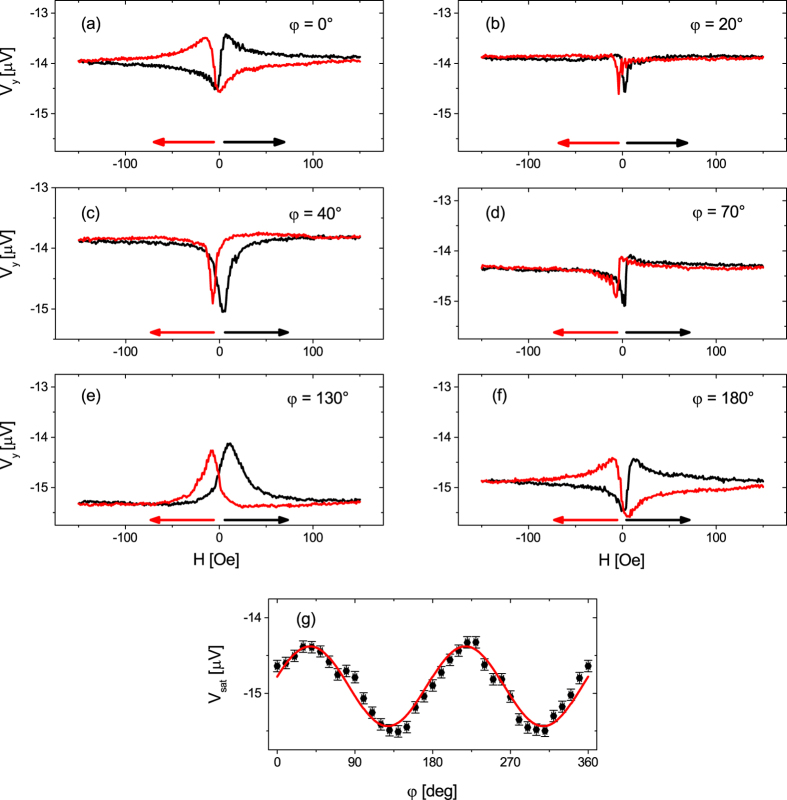
(**a**–**f**) Measurement of *V*_y_ against the magnetic field in a Py film on a MgO substrate. The temperature difference Δ*T* = 30 K was kept constant along the x direction (*φ*_T_ = 0°). The in-plane angle *φ* of the external magnetic field was varied. Data from *φ* = 0° to 180° are shown, since the sin 2*φ* symmetry repeats the course for *φ* ≥ 180°. (**g**) The voltage *V*_sat_ for each *φ* was averaged in the range of 140 Oe ≤ |*H*| ≤ 150 Oe and plotted against *φ*, showing the theoretical predicted sin 2*φ* dependence (Eq. (2)).

Next, the sample was kept at a constant temperature difference of Δ*T*_x_ = 30 K, so the cold side was kept at 293 K and the hot side at 323 K. Sweep measurements were recorded for 0° ≤ *φ* ≤ 360° and six exemplary chosen curves in the range of 0° ≤ *φ* ≤ 180° are shown in Fig. 4(a–f). As before, *V*_y_ saturates for high magnetic fields but shows differently shaped extrema, depending on *φ*. Figure 4(a) shows the same data set as Fig. 3(e) with the appearance of a minimum and a maximum. Increasing *φ* to 20° (Fig. 4(b)) changes the signal at the low magnetic regime into a minimum for both branches with low intensity but similar shape. For *φ* = 40° (Fig. 4(c)) the intensity of these minima increases until for *φ* = 70° (Fig. 4(d)) the curves have changed their shape into a minimum and maximum again. But in contrast to Fig. 4(a) both branches have the same progression, thus, the magnetization reversal process is independent of the sweep direction of the magnetic field. For *φ* = 130° (Fig. 4(e)) large, clearly separated maxima can be observed, which, in case of *φ* = 180° (Fig. 4(f)), form a similar curve as for *φ* = 0°. For angles larger than *φ* = 180° the curves from the range 0° ≥ *φ* ≥ 180° are repeated.

**Figure 5:**
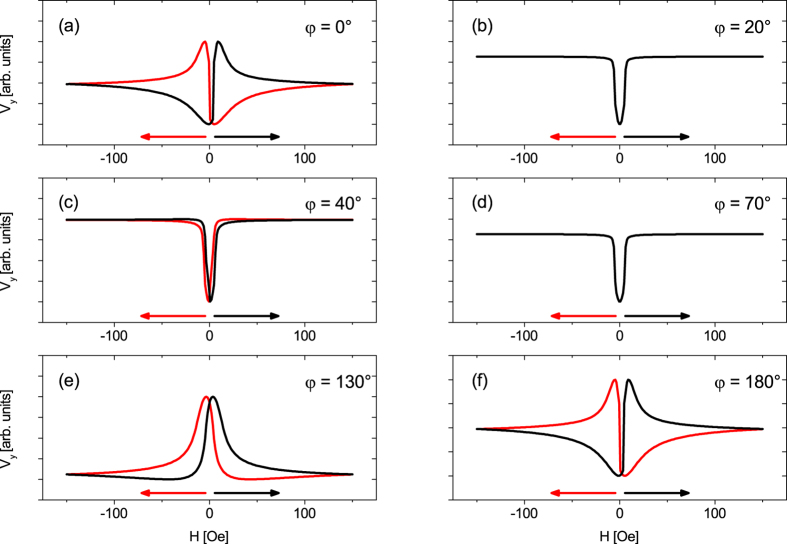
Subsequent simulations based on the Stoner-Wohlfarth model, described in SI chapter III, fit the experimental data of Fig. 4.

The small signals of both branches for *φ* = 20°, 70° indicate magnetic easy axes in these directions^22^. The appearance of two magnetic easy axes tilted by 50° can be explained by the non-parallel superposition of a uniaxial and a cubic magnetic anisotropy (see SI chapter III including refs 39, 40, 41, 42, 43, 44, 45, 46, 47, 48, 49). Furthermore, the experimental data can be fully understood and explained by simulations based on the Stoner-Wohlfarth model taking the geometry of the electromagnets into account (Fig. 5, see SI chapter III including refs 50, 51, 52, 53, 54, 55, 56, 57). The signals for *φ* = 20°, 70° are the same in the simulations (Figs. 5(b,d)), whereas in the experiment they are not. Furthermore, the experiment observes a larger shift between the up and down trace, but beside of the mentioned issues, the simulations fit the experimental data qualitatively well.

Meier *et al*.^22^ split the curves into a symmetric and antisymmetric part. A systematically observed antisymmetric part would indicate an ANE induced by an unintended out-of-plane 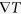. Using this method for the data from Fig. 4 does not show any systematic dependence of the antisymmetric contribution on the direction of the external magnetic field as it would be the case for the ANE. Therefore, we can exclude any unintended out-of-plane 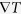 for the new setup, as we could for our other thermal setups^22^,^26^. The small non-systematic antisymmetric contributions can rather be explained by a non-perfect antisymmetric magnetization reversal process for some magnetic field directions due to an interplay of the magnetic anisotropy and field contributions mentioned in the SI chapter III.

Not only the shape of the curves but also the saturation voltage depends on *φ*. All saturation voltages for |*H*| ≥ 140 Oe of each *φ* were averaged, plotted vs. *φ* and after subtraction of a linear temperature drift, *V*_sat_ shows a clear sin 2*φ* dependence (Fig. 4(g)). *V*_sat_ oscillates around an offset voltage of ≈−15.0 *μ*V, which originates from the ordinary thermovoltage, which is described in Eq. (1) by *S*_+_. Small deviations of *V*_sat_ to the fit can be found around *φ* = 90°, 270°, but an analysis of *V*_sat_ − *V*_sin2*φ*_ reveals no systematical higher order measurement artefacts. Since the oscillation of Fig. 4(g) confirms the sin 2*φ* dependence as predicted for the PNE by Eq. (2), further measurements in the rotation measurement mode for different Δ*T* are conducted to track down the PNE.

**Figure 6:**
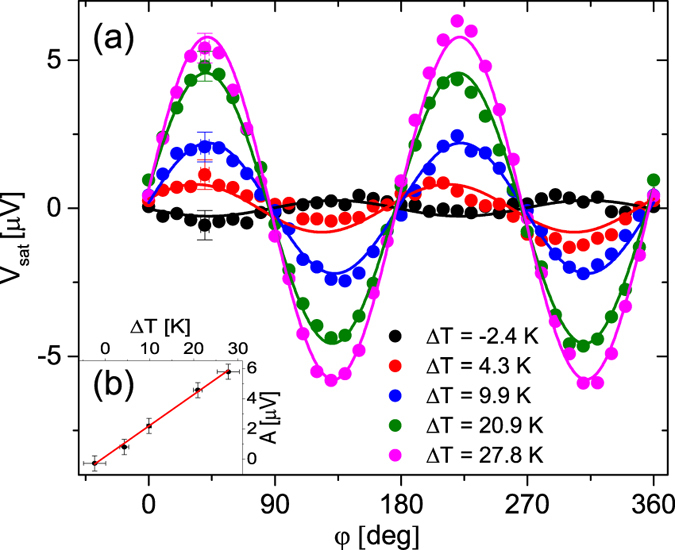
(**a**) An external magnetic field of 200 Oe was rotated in-plane, keeping 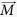 saturated and aligned along *φ*. The measurement was repeated for increasing Δ*T* at *φ*_T_ = 0° and fitted with *V*_sat_ = *y*_0_ + *A* sin(2*φ* − *φ*_0_). The uncertainties *δφ* and *δV*_sat_ are only shown for the data points at *φ* = 40° for reasons of better overview. (**b**) The fit parameter *A* as a function of Δ*T* shows again the linear dependency with respect to Δ*T*.

Five measurements were conducted for each Δ*T*, averaged and plotted in Fig. 6(a). All curves show the expected sin 2*φ* oscillation so that based on Eq. (2) the data were fitted by *V*_sat_ = *y*_0_ + *A* sin 2(*φ* − *φ*_0_), very well confirming the agreement between the data and the theory of the PNE. Furthermore, plotting the amplitude *A* of the fits vs. Δ*T* again shows the expected proportionality between the PNE and Δ*T* (Fig. 6(b)).

### Phase shift of ∇*T* angular dependence of the AMTP and PNE

**Figure 7:**
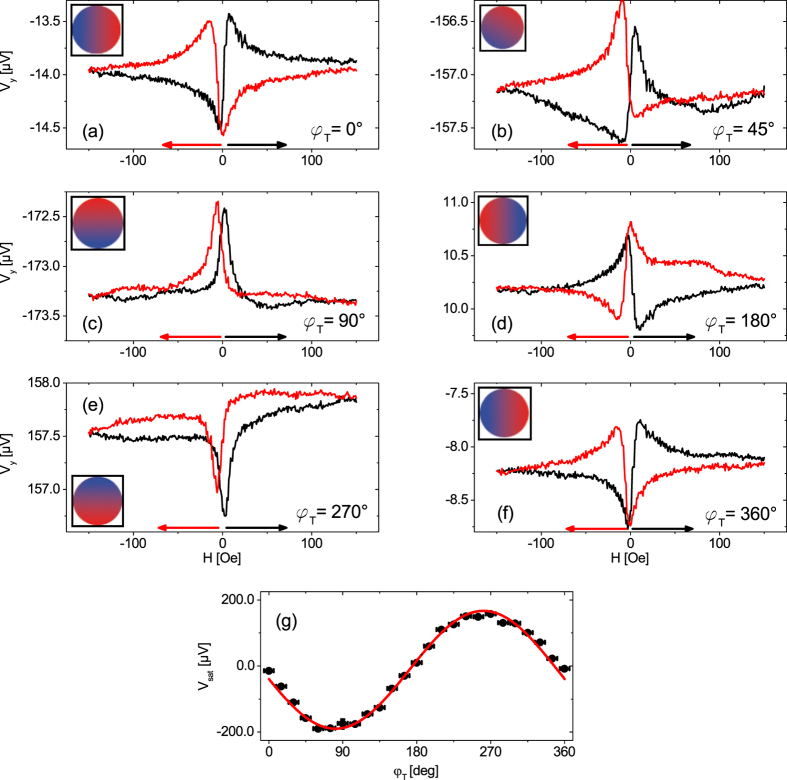
(**a**–**f**) *V*_y_ as a function of the magnetic field for *φ* = 0° and various *φ*_T_. The insets indicate the directions *φ*_T_ of the thermal gradients. The sample was kept at a base temperature of 308 K with Δ*T* = 30 K. (**g**) The voltage *V*_sat_ was averaged as described for Fig. 4(g) and plotted against *φ*_T_. The data show a sin *φ*_T_ dependence attributed to *S*_+_.

Next, the angle of the thermal gradient, *φ*_T_, was continuously increased by 15° and sweep measurements were conducted for *φ* = 0°. Each curve again shows a saturation voltage for high magnetic fields and two extrema close to each other at around 0 Oe (Fig. 7(a–f)). In case of (a) the voltage measurement is carried out perpendicular to the thermal gradient, thus, the signal originates from the PNE 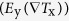. In case of (c) the voltage measurement is conducted parallel to the thermal gradient because it was rotated to *φ*_*T*_ = 90°. Here, the voltage signal is attributed purely to the AMTP, since this effect needs a longitudinal 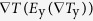.

**Figure 8:**
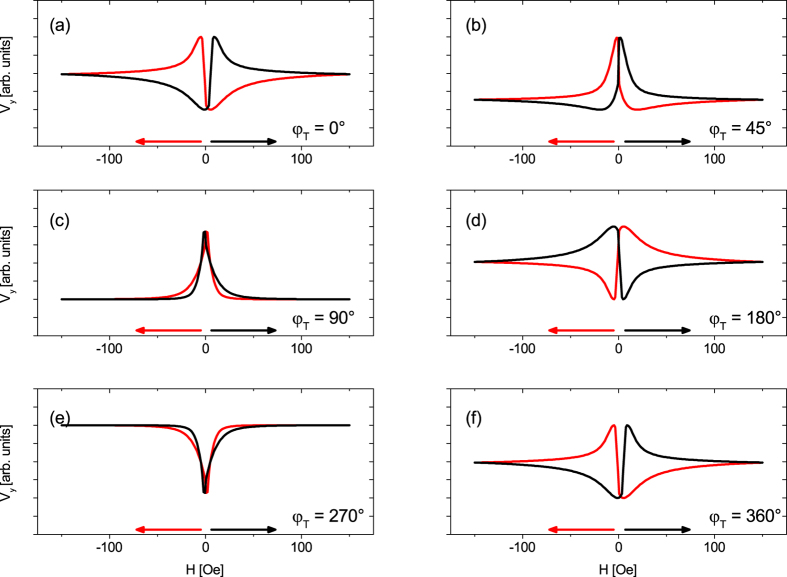
The data of the sweep measurements for rotated 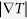 (see Fig. 7) can be simulated with the same model as used in Fig. 5.

The results for 0° < *φ*_T_ < 90° consist of a superposition of the PNE and the AMTP since for these *φ*_T_, 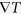 consists of a x and y component. This qualitative change in the signal can also be seen in the voltage features for low magnetic fields. Figure 7(a) shows the same signal progression as described in section B, with the formation of a minimum before crossing 0 Oe. Increasing *φ*_T_ now suppresses this minimum before the zero crossing point until for *φ*_T_ = 90° only two sharp maxima are shaped. Due to the rotation of 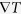 the relative orientation of 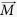 with respect to 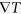 changes for different *φ*_*T*_, thus, leading to changing contributions of the PNE and AMTP to the measured voltage signal. Again, the trace of the voltage signal can be fairly simulated as can be seen in Fig. 8.

Figure 7(g) shows the saturation voltages of Fig. 7(a–f) vs. *φ*_T_. In contrast to Fig. 4(g), where the oscillation of *V*_sat_(*φ*) is only due to the PNE, Fig. 7(g) identifies the contribution of the ordinary, magnetic field independent Seebeck effect *V*_sat_(*φ*_*T*_), expressed by *S*_+_ in Eq. (1). Since *V*_y_ is measured, the rotation of 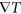 leads to a sin *φ*_T_ shaped projection of 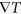 on the y axis, resulting in a sine shaped *V*_y_ signal. The nonmagnetic Seebeck signal is three orders of magnitude higher than the one of the PNE, while the magnetic field dependent part of the AMTP is expected to be of the same order of magnitude than the PNE.

**Figure 9:**
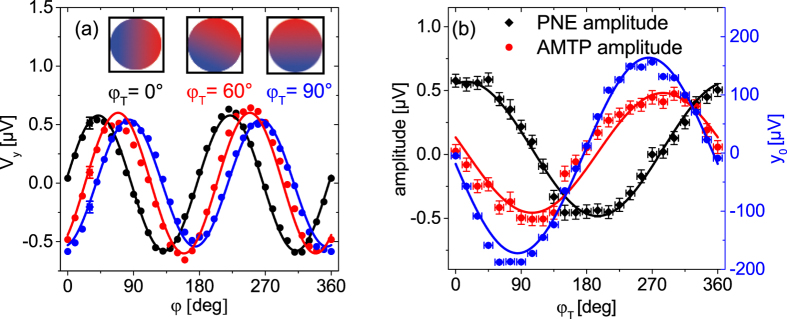
(**a**) *V*_y_ was measured in saturation (200 Oe) while rotating 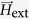 for different 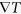 angles *φ*_T_. The uncertainties *δφ* and *δV*_*y*_ are only shown for the data points at *φ* = 30° for reasons of better overview. Again, the insets visualize the directions *φ*_T_ of the thermal gradients. Increasing *φ*_T_ results in a phase shift in the rotation measurement and further shifts the offset position *y*_0_ from −4.83 *μ*V (*φ*_T_ = 0°) over −187.6 *μ*V (*φ*_T_ = 60°) to −187.1 *μ*V (*φ*_T_ = 90°). The phase shift indicates a superposition of PNE and AMTP. Therefore, the data were fitted with a cos 2*φ* (AMTP) and a sin 2*φ* (PNE) superposition. (**b**) The amplitudes of the cos 2*φ* and the sin 2*φ* contributions as well as the offset y_0_ in the rotation measurement were plotted against *φ*_T_ showing the expected cos- (PNE), sin- (AMTP) and sin- (ordinary Seebeck effect) dependence on *φ*_T_.

For the direct comparison of the different AMTP and PNE contributions, rotation measurements for 0° ≤ *φ*_T_ ≤ 360° were conducted. Figure 9(a) shows rotation measurements for three different *φ*_T_, with offset voltages *y*_0_ substracted. As described above, the oscillating signal of *V*_y_ at *φ*_T_ = 0° originates purely from the PNE and the oscillation of *φ*_T_ = 90° purely from the AMTP. Since for all *φ*_T_ in between we obtain a superimposed signal of both, the rotation measurements for all *φ*_T_ were fitted with


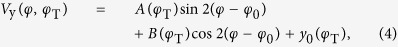


with


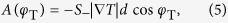



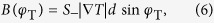



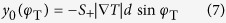


based on Eqs (1) and (2). Here, the fit parameters *A* and *B* indicate the amplitudes of the PNE and AMTP, respectively. *d* is the distance of the electric contacts and *y*_0_ is the offset in *V*_y_, which mirrors the superpositioned ordinary Seebeck effect of the Au bonding wires and Py film, expressed as *S*_+_. When *V*_y_ is plotted vs. *φ*, Fig. 9(a) shows the superposition of all effects, which leads to a phase shift of the measured signal for *φ*_T_ > 0°, described by Eq. (3). The sin 2*φ* dependence (for *φ*_T_ = 0°), expected for the PNE (Eq. (2)) is shifted to a −cos 2*φ* dependence (for *φ*_T_ = 90°), predicted for the AMTP (Eq. (1)).

In addition to the detected phase shift in the resulting signal, the change of the PNE (AMTP) ratio for each *φ*_T_ can be revealed by plotting the fit amplitude *A (B*) vs. *φ*_T_ (Fig. 9(b)). The result clearly shows a cosine (PNE) and a sine (AMTP) dependence of the amplitudes on *φ*_T_ as determined by Eqs (5) and (6). The resulting cosine and sine fit functions result in a PNE amplitude of (0.53 ± 0.05) *μ*V and an AMTP amplitude of (−0.47 ± 0.05) *μ*V. Within the measurement uncertainty the absolute value of the magnitudes of both effects are the same as it was expected from Eqs (5) and (6). Additional to the amplitudes, plotting *y*_0_ vs. *φ*_*T*_ gives a sine function as Eq. (7) predicts.

With these findings we can determine the thermovoltages 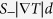 and 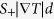. Averaging the absolute values of the amplitudes of *A* and *B* results in


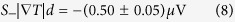


and the amplitude of *y*_0_ gives


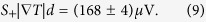


To separate the thermopower of the Au bonding wires from the conventional thermopower of the Py thin film, *S*_+_ has to be regarded as an effective Seebeck coefficient *S*_+_ = *S*_*eff*_ = *S*_*Py*_ − *S*_*Au*_ (see SI, chapter IV with refs 58,59), taking the literature values of *S*_*Py*_ and *S*_*Au*_ into account. This allows the estimation of the applied temperature difference between the bonding wires to be Δ*T* = 26.7 K, which agrees with the applied temperature difference of 30 K. Δ*T* is used to calculate 
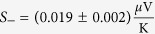
 which is then compared to the conventional Seebeck coefficient of the pure Py thin film. The relative change of the anisotropic Seebeck coefficient of Py, Δ*S*, is then given by


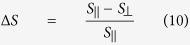



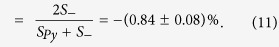


This calculation shows that the magnetothermopower perpendicular to the magnetization is 0.84% stronger than parallel to the magnetization. The rotation of 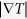 was used to sucessfully seperate PNE from AMTP measurements, which is observed by the subsequent shift of a sin- to a cos-dependence of the magnetic field rotation measurement.

## Conclusion

In conclusion, a novel setup was realized, which allows a well-defined rotation of an in-plane thermal gradient by superposition of two perpendicular thermal gradients of variable strength. Thus, the simultaneous measurement of the AMTP and PNE has been made possible. The functionality of the setup was demonstrated and analyzed by an infrared camera and could further be verified by the subsequent electric analysis of magnetothermopower effects in a permalloy thin film on MgO(001). First, the proportionality dependency of the PNE to the temperature difference was shown. Second, a sweep of the external magnetic field was conducted for different angles and spatial fixed 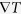, showing a repetition of the voltage signal for angles larger than 180°. Plotting the saturation voltages vs. the magnetic field angle *φ* shows a sin 2*φ* dependency, verifying the theoretical predictions. By only rotating a high magnetic field, these sin 2*φ* oscillations can be measured directly. Measuring them for rotated 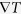 leads to a phase shift until for *φ*_*T*_ = 90° the sin 2*φ* oscillation of the magnetic field angular dependence is shifted to a cos 2*φ* oscillation. This shift is due to a superposition of the PNE and AMTP and is the proof for a successful and controlled rotation of 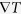. It further enables the splitting of the measured signal into *φ*_*T*_ dependent contributions of the PNE, AMTP and ordinary Seebeck effect. After excluding the thermovoltage contribution of the Au bonding wires, the thermovoltages parallel and perpendicular to the magnetization of Py can be estimated by using *S*_*Py*_, *S*_−_ and Δ*T*


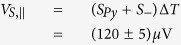


and


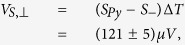


resulting in a relative magnitude of the anisotropic magnetothermopower of Δ*S* = −(0.84 ± 0.08)%.

After having proved the rotation of 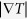 with respect to the crystal structure, this setup is a promising tool to establish this method in future spin caloric experiments such as detailed anisotropy investigations of the spin Nernst magnetothermopower.

## Additional Information

**How to cite this article:** Reimer, O. *et al*. Quantitative separation of the anisotropic magnetothermopower and planar Nernst effect by the rotation of an in-plane thermal gradient. *Sci. Rep.* **7**, 40586; doi: 10.1038/srep40586 (2017).

**Publisher's note:** Springer Nature remains neutral with regard to jurisdictional claims in published maps and institutional affiliations.

## Supplementary Material

Supplementary Information
