# Supplementary material for: Quantitative separation of the anisotropic magnetothermopower and planar Nernst effect by the rotation of an in-plane thermal gradient
Source: Sci Rep. 2017 Jan 17;7:40586. doi: 10.1038/srep40586 (PMC5240136; doi:10.1038/srep40586)
Supplement: Supplementary Information [file srep40586-s1.pdf]

# - Supplementary Information -
Quantitative separation of the anisotropic magnetothermopower and planar Nernst effect by the rotation of an in-plane thermal gradient

Oliver Reimer[1], Daniel Meier[1], Michel Bovender[1], Lars Helmich[1], Jan-Oliver Dreessen[1], Jan Krieft[1], Anatoly S. Shestakov[2], Christian H. Back[2], Jan-Michael Schmalhorst[1], Andreas Hütten[1], Günter Reiss[1], and Timo Kuschel[1,3]

[1] *Center for Spinelectronic Materials and Devices, Department of Physics, Bielefeld University, Universitätsstraße 25, 33615 Bielefeld, Germany*

[2] *Institute of Experimental and Applied Physics, University of Regensburg, Universitätsstraße 31, 93040 Regensburg, Germany*

[3] *Physics of Nanodevices, Zernike Institute for Advanced Materials, University of Groningen, Nijenborgh 4, 9747 AG Groningen, The Netherlands*

In the first part of the supplementary information we derive a theoretical description of the magnetothermopower effects and an equation optimized for our measurement geometry. The second part describes the details of the experimental setup and its calibration. It is followed by the optical investigation via an infrared camera of different substrates. In the third part, we further show that the electric measurements can be very well fitted by simulations based on the Stoner-Wohlfarth model that are supported by x-ray diffraction measurements of the used sample. The fourth chapter contains the correction of the measured thermopower regarding the thermopower contribution of the Au bonding wires.

THEORETICAL BACKGROUND OF MAGNETOTHERMAL EFFECTS

The anisotropic magnetoresistance (AMR) describes the difference of the electric resistivity parallel and perpendicular to the magnetization direction of a ferromagnetic conductor[1]. The overlap of atom orbitals changes with the direction of the magnetization due to spin-orbit coupling. This affects the scattering cross section and, therefore, the electric resistivity, which is typically reduced for a magnetization direction perpendicular to the electric current. Thus, the measured voltage across a ferromagnet depends on the directions of current and magnetization. Assuming a current density along x, $|\vec{J}| = J_{\mathrm{x}}$ (Fig. 1 (a)), the longitudinal electric field E_{x} depends on the electric field components parallel and perpendicular to the magnetization. Following Thompson *et al.*, these components $E_{||}$ and $E_{\perp}$ can be described by J, the resistivity ρ and the angle φ between the magnetization and the x-axis[2]. One obtains

$$E_{||} = \rho_{||}\, J_{\mathrm{x}}\, \cos\varphi \tag{1}$$

$$E_{\perp} = \rho_{\perp}\, J_{\mathrm{x}}\, \sin\varphi \quad . \tag{2}$$

The projections of $E_{||}$ and $E_{\perp}$ onto the x-axis are

$$E_{\mathrm{x},||} = E_{||}\, \cos\varphi = \rho_{||}\, J_{\mathrm{x}}\, \cos^2\varphi \tag{3}$$

$$E_{\mathrm{x},\perp} = E_{\perp}\, \sin\varphi = \rho_{\perp}\, J_{\mathrm{x}}\, \sin^2\varphi \tag{4}$$

leading to

$$E_{\mathrm{x}} = E_{\mathrm{x},||} + E_{\mathrm{x},\perp} \tag{5}$$

$$= \rho_{||}\, J_{\mathrm{x}}\, \cos^2\varphi + \rho_{\perp}\, J_{\mathrm{x}}\, \sin^2\varphi \quad . \tag{6}$$

Using $\sin^2\varphi = 1 - \cos^2\varphi$ and $\cos^2\varphi = \frac{1+\cos 2\varphi}{2}$ the longitudinal electric field describing the longitudinal AMR results in

$$E_{\mathrm{x}} = \left(\frac{\rho_{||} + \rho_{\perp}}{2} + \frac{\rho_{||} - \rho_{\perp}}{2} \cos 2\varphi \right) J_{\mathrm{x}} \quad . \tag{7}$$

A similar consideration leads to the expression for the electric field E_y, transverse to the current density J_x. Here, the y-components of $E_{||}$ and $E_\perp$ have to be considered. One obtains

$$E_y = E_{y,||} + E_{y,\perp} \tag{8}$$
$$= (\rho_{||}\, J_x - \rho_\perp\, J_x) \cos\varphi \, \sin\varphi \quad . \tag{9}$$

With $\cos\varphi \, \sin\varphi = \frac{1}{2}\sin 2\varphi$,

$$E_y = \frac{\rho_{||} - \rho_\perp}{2} \sin 2\varphi \, J_x \tag{10}$$

describes the transverse electric field, also called transverse AMR or planar Hall effect (PHE).

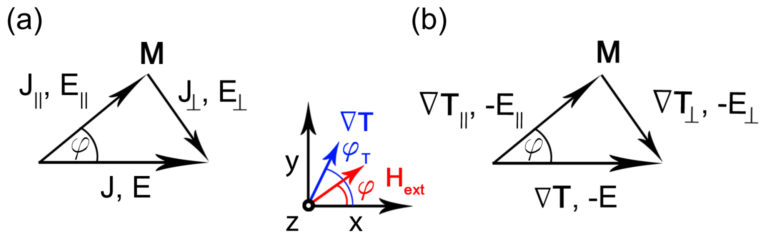

FIG. 1. (a) When an electric current is driven through a ferromagnetic conductor along x, the electric resistance parallel and perpendicular to M is different due to the AMR. The different electric fields parallel and perpendicular to M result in different electric fields along the x- and y-direction strongly depending on the angle between the electric current and M. (b) Thermal analogon to (a): A temperature gradient is the driving force of an electric current along x. Due to the anisotropic orbitals of the atoms, the parallel and perpendicular Seebeck coefficients differ from each other. Thus, the measured voltage is strongly dependent on the direction of the magnetization.

If J_x is substituted by a thermal gradient along the x-direction (∇T_x), the Seebeck effect drives an electric current through the sample, leading to thermal equivalent effects of the above described current-driven effects. The Seebeck coefficient is anisotropic for the directions parallel or perpendicular to the magnetization (Fig. 1 (b)). In case of a longitudinal measurement in an open circuit geometry the anisotropic magnetothermopower (AMTP) as the thermal counterpart of the AMR (Eq. 7) will lead to an electric field along the x-direction

$$E_x = -\left(\frac{S_{||} + S_\perp}{2} + \frac{S_{||} - S_\perp}{2} \cos 2\varphi\right) \nabla T_x \quad . \tag{11}$$

In case of a transverse measurement the planar Nernst effect (PNE) as the thermal counterpart of the PHE (Eq. (10)) will induce an electric field along the y-direction[3]

$$E_y = -\frac{S_{||} - S_\perp}{2} \sin 2\varphi \, \nabla T_x \quad . \tag{12}$$

Since this work demonstrates the rotation of ∇T in the xy-plane, a superposition of the AMTP and PNE is expected for other directions of ∇T than the x- or y-axis. Assuming a measurement of the AMTP along the x-direction for rotated ∇T, Eq. (11) adjusts to

$$E_x = -\left(\frac{S_{||} + S_\perp}{2} + \frac{S_{||} - S_\perp}{2} \cos 2\varphi\right) |\nabla T| \cos\varphi_T \quad , \tag{13}$$

with the angle φ_T between ∇T and the x-axis (Fig. 1). Similarly, Eq. (12) for the PNE changes to

$$E_y = -\frac{S_{||} - S_\perp}{2} \sin 2\varphi \, |\nabla T| \cos\varphi_T \quad . \tag{14}$$

Now, it has to be taken into account that the AMTP is measured along the y-axis and the angles in Eq. (13) are defined with respect to the x-axis. Therefore, an angle phase shift has to be introduced which considers that the (longitudinal) AMTP is measured along the y-axis. Keeping the angles defined along the x-direction shifts E_{y} by 90°, thus leading to

$$\begin{aligned} E_{\mathrm{y}} &= -\left(\frac{S_{||}+S_{\perp}}{2}+\frac{S_{||}-S_{\perp}}{2}\cos\left(2(\varphi-90^{\circ})\right)\right)|\nabla T|\,\cos(\varphi_T-90^{\circ}) \\ &= -\left(\frac{S_{||}+S_{\perp}}{2}-\frac{S_{||}-S_{\perp}}{2}\cos 2\varphi\right)|\nabla T|\,\sin\varphi_T \quad . \end{aligned} \tag{15}$$

Hence, Eq. (15) describes the longitudinal (AMTP) and

$$E_{\mathrm{y}} = -\frac{S_{||}-S_{\perp}}{2}\sin 2\varphi\,|\nabla T|\,\cos\varphi_{\mathrm{T}} \tag{16}$$

the transverse magnetothermopower (PNE) along the y-direction. Since the experiment will measure the AMTP and PNE simultaneously, the superpositioned electric field along the y-direction is described by

$$E_{\mathrm{y}} = -(S_{+}\sin\varphi_{\mathrm{T}} + S_{-}\sin(2\,\varphi-\varphi_{\mathrm{T}}))|\nabla T| \tag{17}$$

with $S_{+} = \frac{S_{||}+S_{\perp}}{2}$ and $S_{-} = \frac{S_{||}-S_{\perp}}{2}$, showing that a variation of φ_{T} leads to a phase shift in E_{y}.

EXPERIMENTAL SETUP

Applying a magnetic field

For thermomagnetic and spin caloric measurements an external, rotatable magnetic field is eminently useful to identify the occurring transport phenomena by its symmetry. For this purpose two pairs of electromagnets with iron cores are arranged next to the sample holders (Fig. 2 a)). The distance of the iron cores can be varied, in order to be flexible for different sample sizes.

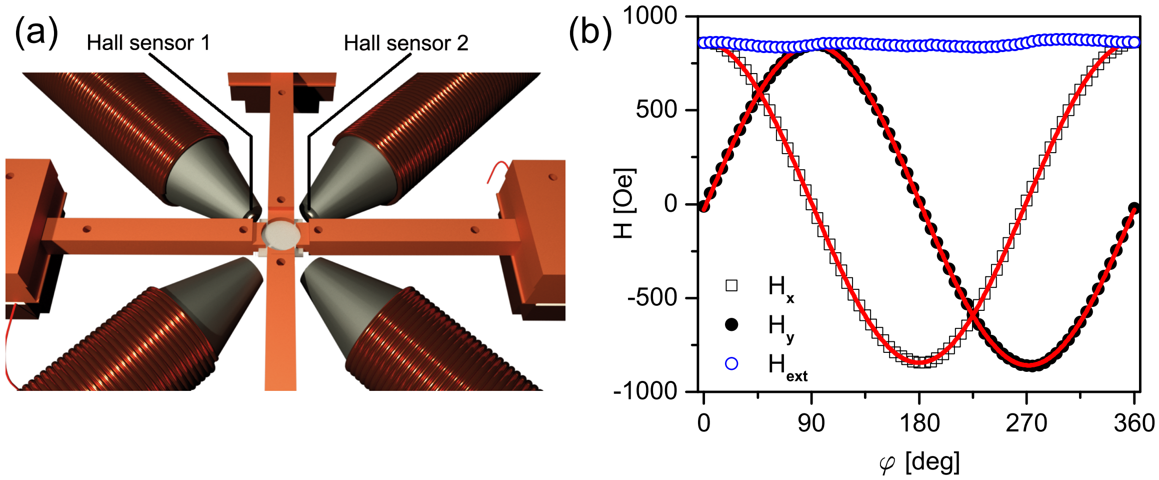

FIG. 2. a) Two electromagnet pairs provide the desired $\vec{H}_{\mathrm{ext}}$ along φ which is measured via two Hall sensors at the pole caps. b) The magnetic field rotation is verified by fitting the x- (y-) component of the applied magnetic field with a cosine (sine) function. The superposition of $\vec{H}_{\mathrm{x}}$ and $\vec{H}_{\mathrm{y}}$ results in $\vec{H}_{\mathrm{ext}}$ which is constant within 1.4%.

The superposition of the fields of both magnetic axes gives a net magnetic field with a selectable angle φ. Hall sensors (Projekt Elektronik, AS-NTP-Flex) are attached to the pole caps of one iron core of each magnetic axis. After

calibrating its signal to the magnetic field at the sample position the simultaneous measurement of $\vec{H}_{\mathrm{ext}}$ during the experiment is possible. In this manner magnetic fields up to $\approx$ 900 Oe can be reached. The successful magnetic field rotation is proven by measuring the x- and y-component of $\vec{H}_{\mathrm{ext}}$ and fitting them to a cosine (sine) for H_{x} (H_{y}) (Fig. 2 b)). The calculated resulting $\vec{H}_{\mathrm{ext}}$ stays constant within a standard deviation of 12 Oe at a mean value of 853 Oe.

Electric contacting

The setup allows the installation of four micro-probe measurement stages to electricly contact the samples via, e.g., contact needles. For the lowest background noise (RMS $\approx$ 50 nV) the samples were bonded with 25 μm thin Au wires whose ends were glued to Au probes by silver paste. The electric signal is then detected by a nanovoltmeter (Keithley 2182A).

Detecting the temperature profiles

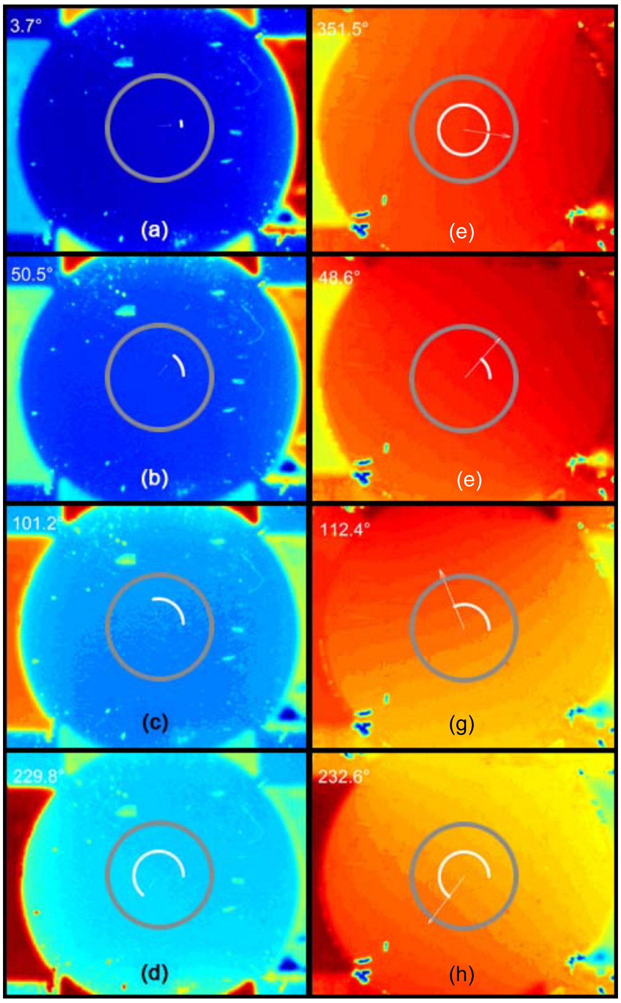

FIG. 3. Thermographic pictures taken for (a)-(d) MgO and (e)-(h) Cu for (a), (e) $\varphi_{\mathrm{T}} = 0°$; (b), (f) $\varphi_{\mathrm{T}} = 45°$; (c), (g) $\varphi_{\mathrm{T}} = 105°$; (d), (h) $\varphi_{\mathrm{T}} = 225°$. All substrates and sample holder were coated with high absorbing clustered Au particles. Small deviations between the applied φ_{T} and the calculated average angle of up to 6° can be detected due to surface defects, inhomogenous ∇T, different thermal conductivities or a relative rotation between the setup and the infrared camera.

In addition to the electric temperature detection, a FLIR SC7000 infrared camera is used to detect the temperature profiles of the sample. A quantitative comparison between various substrates despite their different emissivities is

allowed by depositing a high absorbing, thin layer of clustered Au particles under nitrogen atmosphere on each substrate, following Lang *et al.*[6]. The vertical mount of the IR camera 0.5 m above the sample allows the optical detection of the heat distribution directly at the sample surface. In contrast to the electric temperature detection by the PT1000 thermometers this method excludes any temperature losses via heat conduction, e.g., due to the Cu clamps. These data are used to quantify parameters like the magnitude of temperature gradient, base temperature and angle of the applied ∇T as follows: First, a circular region of interest (ROI) is defined where data should be considered from for the following calculations and which excludes possible surface defects (grey circles in Fig. 3). Second, for each data point in the ROI the local temperature gradient in x- and y-direction is calculated which leads to a determination of an average temperature gradient. These quantities are used to identify the average angle under which ∇T is applied (symbolized by the white arcs in Fig. 3). Third, the vectorial temperature profile along the calculated angle is used for a linear fit whose origin is the temperature at the center of the ROI (base temperature of sample) and whose slope is equal to ∇T.

As a first proof of the successful rotation of ∇T, copper- (Cu-) and MgO- substrates were thermographically investigated. Figure 3 shows pictures taken from the infrared camera for a MgO and Cu substrate, after applying ∇T at $\varphi_T = 45°, 105°, 225°$ and 360°. Although a deviation between the applied φ_T and the calculated average output angle is observed, the rotation of ∇T could be controlled in this measurement within an uncertainty of 6°.

SUPERPOSITION OF MAGNETIC ANISOTROPIES

The appearance of two magnetic easy axes (MEA, $\varphi = 20°, 70°$) can be explained by the superposition of a uniaxial (UMA) and a fourfold in-plane cubic magnetic anisotropy (CMA). A UMA can be attributed to, e.g., surface steps[7], oblique growth[8], substrate shape[9] or dangling bonds[10]. For example, Fe/MgO(001) systems were used to manipulate the UMA in terms of strength and orientation with different deposition techniques[11,12] showing the likelihood of forming a UMA during the deposition of thin films, not only for Fe/MgO(001) but also for permalloy thin films on different substrates[13,14]. Due to the crystalline symmetry of cubic magnetic films a CMA is expected to be present for cubic systems[11,12]. X-ray diffraction (XRD) measurements on our Py/MgO(001) sample show a fourfold diffraction pattern at a 2Θ-angle of 44.332° for (111) Bragg reflections (Fig. 4), which can clearly be separated from the MgO(001) signal. Therefore, the presence of a CMA is very likely.

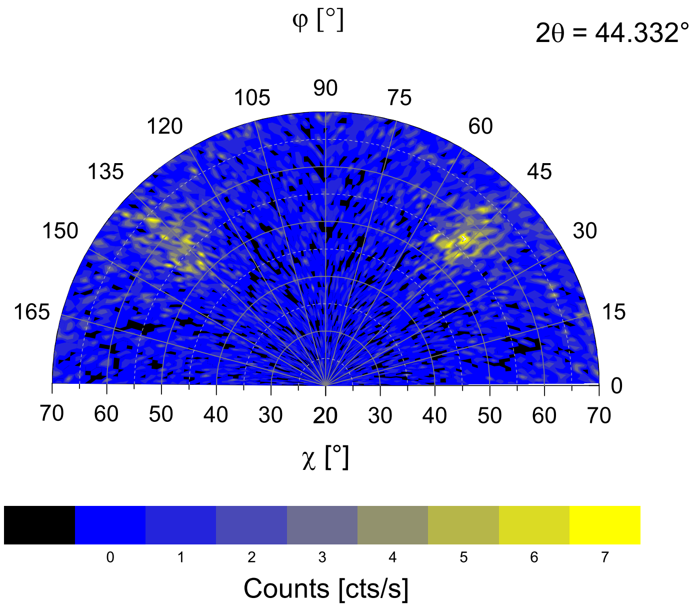

FIG. 4. XRD measurement of Py/MgO(001) at $2\Theta = 44.332°$ shows a fourfold diffraction pattern, indicating a cubic crystal structure.

The magnetic field dependent voltages in Fig. 4 and 7 in III. B and III. C (main paper) have partially asymmetric behavior, although AMTP/PNE traces are known to be symmetric[15–20] to the magnetic field in case of a present

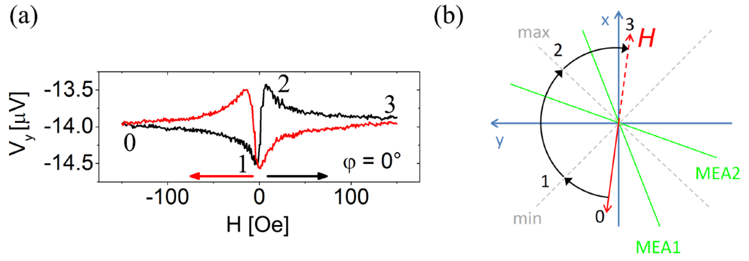

FIG. 5. Explanation of experimental field sweeps: (a) The experimental sweep up trace for $\varphi = 0°$ passes through 4 specific positions, (b) $\vec{M}$ reversal in relation to the experiment at $\varphi = 0°$. MEA1 and MEA2 (green lines) are magnetic easy axes situated around 20° and 70°, respectively.

UMA. To resolve this situation different simulations were conducted and described in the following. For Eqs. (15), (16) it is implicitly assumed that the magnetization vector $\vec{M}$ coincides with the magnetic field $\vec{H}$ which means that $\varphi_{M0} = \varphi$, with φ_{M0} as the angle of equilibrium position of $\vec{M}$. This assumption works well when the applied magnetic field is at least one order of magnitude stronger than the magnetic anisotropy or any parasitic magnetic field $\vec{H}_p$ contribution in the system[21]: $|H| >> \frac{C_i K_i}{M_s}, |H_p|$, where K_i is some anisotropy constant with the dimension of J/cm^3, M_s is the saturation magnetization and C_i is a dimensionless constant of the order of unity (depending on the type of anisotropy). However, this assumption is fulfilled only for external magnetic fields greater than 100 Oe. Thus, an adequate model for calculations of φ_{M0} for lower external magnetic fields is necessary, in order to reproduce complete field sweeps of the experimental AMTP/PNE traces.

First of all, using $V_y = -E_y d$ (d is the distance between measuring contacts) Eq. (17) can be rewritten as

$$V_y(H) = d\, S_- \,|\nabla T| \sin(2\varphi_{M0}(H) - \varphi_T) \quad , \tag{18}$$

where the summand with $S_+ = \frac{S_\parallel + S_\perp}{2}$ is neglected, since it does not depend on $\vec{H}$ and produces only an offset in the magnetic field sweep measurements. In further calculations and in Figs. 5 and 8 of the main paper normalized AMTP/PNE traces are shown ($d\, S_- \,|\nabla T| = 1$). The experimental data of magnetic field sweeps (Fig. 4, main paper) hint to the presence of a magnetic anisotropy. Magnetic easy directions (magnetic easy axes MEA1 and MEA2) are situated around $\varphi_{min1} = 20°$ and $\varphi_{min2} = 70°$, since the signal amplitudes are smallest in these directions. This kind of anisotropy could result from the combination of UMA and CMA of the sample which is not parallelly aligned as mentioned previously[11,12,22,23].

As a first suggestion for the rotation of $\vec{M}$, but later proven by model C, the shape of the experimental curves can be understood with the help of Fig. 5 for the sweep up curve at $\varphi = 0°$ and $\varphi_T = 0°$. According to Eq. (18) the maximum and minimum of the signal appears for $\varphi_{M0} = 45°$, 225° and $\varphi_{M0} = 135°$, 315° respectively (dashed, gray lines in Fig. 5(b)). Point **0** is the fully saturated state of the sample, when $\vec{M}$ is aligned with the field $\vec{H}$ (corresponding to negative values of $\vec{H}$). When the absolute field value of $\vec{H}$ goes down, $\vec{M}$ tends to rotate off the direction of the external magnetic field and $\vec{M}$ passes through the direction of minimum **1** (despite it is expected to rotate in the opposite direction, closer towards MEA1). When the magnetic field switches its direction (towards red dashed arrow) and increases its absolute value, $\vec{M}$ passes through the maximum position **2** trying to align with the magnetic field in direction **3**.

Model A: According to Gurevich *et al.*[21], the in-plane density of magnetic free energy U in the presence of a UMA and CMA for monodomain magnetization reads

$$U = -M_S H \cos(\varphi_M - \varphi) + K_U \sin^2(\varphi_M - \varphi_{UA}) + \frac{K_C}{4} \sin^2 2(\varphi_M - \varphi_{CA}) \quad , \tag{19}$$

where the first term is the Zeeman energy, the second and third terms represent the magnetocrystalline uniaxial and cubic anisotropy energies, M_S is the saturation magnetization, K_U and K_C are the constants describing the strength of the UMA and CMA with angles φ_{UA} and φ_{CA} respectively, and φ_M is an arbitrary direction of the magnetization vector $\vec{M}$. The demagnetization energy is excluded, since its in-plane contribution for the given geometry is negligible. The demagnetization factors are calculated according to Aharoni *et al.*[24] which leads to an effective in-plane demagnetization factor $(N_x - N_y)/4\pi$ of the order of 10^{-6} (effective in-plane demagnetizing

field ≈ 0.01 Oe). Using reasonable values of $K_C = 5 \cdot 10^4$ erg/cm^3 [25], $K_U = 2 \cdot 10^4$ erg/cm^3 [26,27], $\varphi_{CA} = 0°$ and $\varphi_{UA} = 45°$ and without an external magnetic field, the free magnetic energy angular distribution shown in Fig. 6 is calculated. As can be seen, the magnetic easy axes are aligned to the previously mentioned directions 20° and 70°. However, with application of this model[27] only symmetric PNE traces can be obtained, because $\vec{M}$ switches between directions 1 and 2 immediately via multidomain state after inverting $\vec{H}$ (described in Fig. 7(b)). A multidomain state is only supposed to exist in an extremely narrow range around $\vec{H} = 0$ Oe, which is a reasonable assumption for Py thin films.

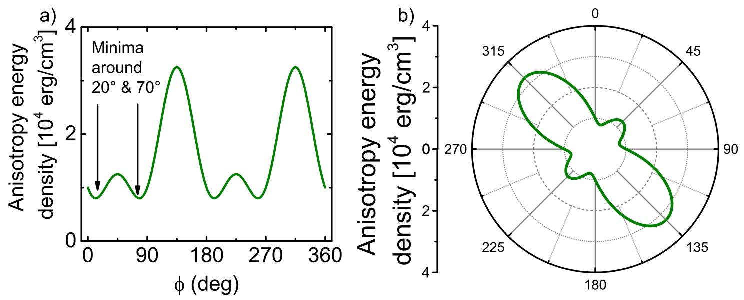

FIG. 6. (a) Energy angular dependence according to Eq. (19) with parameters $K_C = 5 \cdot 10^4$ erg/cm^3, $K_U = 2 \cdot 10^4$ erg/cm^3, $\varphi_{CA} = 0°$ and $\varphi_{UA} = 45°$. Magnetic easy directions are situated around $\varphi_{min1} = 20°$ and $\varphi_{min2} = 70°$; (b) Representation of energy landscape in polar coordinate system.

Model B: A more elaborate investigation with keeping the magnetic prehistory of the sample (hysteresis phenomenon), using multidomain state and including modeling of finite temperatures as well as UMA and CMA was made in MuMax3[28], leading to Fig. 7. In this model it is not possible to conduct simulations for large sample areas (5x5 mm^2), because of the cell number limitation of MuMax3. Instead, an area of 1x1 μm was used. The reduction of area leads to an enhanced contribution of demagnetization energy to anisotropy by 3 orders of magnitude. To make this contribution negligible, M_S has to be reduced twice in comparison to permalloy's value and the anisotropy constants were enlarged by an order of magnitude in comparison to model A. The sweep region was expanded to [-1000; 1000] Oe. Thus, this model only gives a qualitative description. The simulated voltage trace in Fig. 7 (a) gives the antisymmetric behaviour, but yet the behavior of $\vec{M}$ in Fig. 7(b) is very different to the one shown in Fig. 5(b). $\vec{M}$ rotates in direction **1** which corresponds to MEA1, when the field sweep goes up. Then $\vec{M}$ switches its direction by 180° via multidomain state (the region between **1** and **2** in Fig. 7 (a); position 1' is related to $H \approx 0$ Oe) into **2** . The minimum of the voltage trace does not occur because of passing through minimum direction at 135°, but because of the multidomain state, which tends to reduce the absolute value of $\vec{M}$ (AMTP/PNE signal is $\propto |M|^2$). Next, when $\vec{H}$ switches its direction and increases its value, $\vec{M}$ tends to align with the external magnetic field (point **3**). Asymmetric behaviour in systems with CMA, low M_S and large magnetic anisotropy was already observed in other AMTP/PNE experiments[18,20].

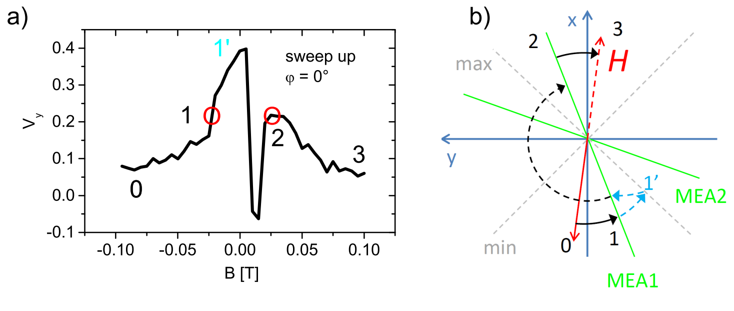

FIG. 7. MuMax3 simulation for sweep up curve: (a) normalized voltage sweep up curve for $\varphi = 0°$; (b) $\vec{M}$ behavior during field sweep up for $\varphi = 0°$.

Model C: Next, the directions of UMA and CMA were analyzed in more detail. The fact that the magnetic hard directions of the CMA coincide with the directions of the magnetic pole caps of the setup, hints to a magnetic anisotropy which rather comes from the magnet system itself than from the sample. In case of a perfect geometry

of the magnet a pure CMA could be expected only due to its symmetry. But with slightly different distances between the opposing magnetic cores and inhomogeneities in the yoke an additional introduction of UMA seems to be possible, just because one magnetic pair might be more preferable for the magnetic flux than the other one. Magnetic hard directions of CMA coincide with the magnetic poles of the magnet, since even when only one pair is used, there is always some remanence magnetization in the second pair. Thus, the fields of both pairs always have to be summed up, so that the superpositioned field direction always tends to stay somewhere between the pole directions.

Now, a parasitic field H_p due to the magnetic yoke is assumed. Because of the direct connection of both magnetic pairs through the toroidal yoke it may lead to a "leakage" of magnetic flux from one pair to the other: The magnetic state of one pair is sensitive to the state of the second pair, while the permeability of the yoke has nonlinear dependence on the magnetic flux passing through it. The magnetic flux of one pair modulates the magnetic transparency of the yoke and influences the behavior of the second pair of poles. With these considerations, the parasitic magnetic field $H_p(\varphi)$ can be written as

$$|H_p(\varphi)| = |H_{pmax}| \, |\sin(\varphi - \varphi_{min1}) \sin(\varphi - \varphi_{min2})| \quad , \tag{20}$$

with $H_{pmax} = 7.5$ Oe as the modulus of parasitic field, $\varphi_{min1} = 20^\circ$ and $\varphi_{min2} = 70^\circ$. $H_p(\varphi)$ is orientated along

$$\varphi_p(H, \varphi) = \varphi \pm 180^o \left(\frac{H_{max} + H}{2H_{max}} \right) \quad , \tag{21}$$

with H_{max} as the amplitude of the applied magnetic field during the sweep (150 Oe). $|H_p(\varphi)|$ (see simulated trace Fig. 8) takes into account the anisotropic character of the magnet system (amplitude of anisotropy). Its angular dependence behaves very similar to energy angular dependence of model A shown in Fig. 6. The phase shift (second summand of angle of parasitic field φ_p in Eq. (21)) takes the "leakage process" and anisotropic character (rotation of $\vec{M}$) into account as well. The sign of the phase shift (+ or $-$) is chosen the way that $\vec{M}$ rotates in the direction of the closest minimum of AMTP/PNE signal (Fig. 5(b)). $\varphi_{CA} = 0^\circ$ of CMA is chosen according to XRD data from Fig. 4 and K_C is the same as in model A.

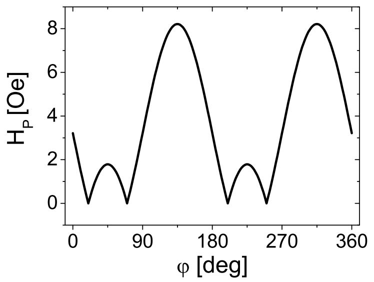

FIG. 8. $|H_p(\varphi)|$ according to Eq. (20) with $H_{pmax} = 7.5$ Oe, $\varphi_{min1} = 20^\circ$ and $\varphi_{min2} = 70^\circ$.

The subsequent calculation for each sweep measurement at a specific angle φ includes the following steps:

1. The parameters $H_{pmax}, \varphi_T, \varphi_{min1}, \varphi_{min2}$ are kept fixed. First, $|H_p(\varphi)|$ is calculated. For each value of the sweep field H, $\varphi_p(H)$ is determined;

2. In Eq. (19) H is substituted with $H_\Sigma(H)$ (effective magnetic field) and φ with $\varphi_\Sigma(H)$ (effective angle). $K_c = 5 \cdot 10^4$ erg/cm^3, $K_u = 0$ erg/cm^3, $\varphi_{ca} = 0^\circ$ are used. In order to find $\varphi_{M0}(H)$ the derivative of Eq. (19) is taken and its root is determined (similar to Shestakov *et al.*[27]). This procedure is done for each value of H. After this step the $\varphi_{M0}(H)$ dependence is known.

3. After inserting the computed dependence $\varphi_{M0}(H)$ into Eq. (18) the normalized curve $V_y(\varphi_\Sigma(H))$ (with $dS_- \, |\nabla T| = 1$) can be constructed and compared with the experimentally determined data.

The calculated traces (Fig. 5 and Fig. 8, see main manuscript) fit the experimental data (Fig. 4 and Fig. 7, see main manuscript) qualitatively well, despite the use of a relatively simple model.

CORRECTION OF OBSERVED THERMOPOWER REGARDING AU BONDING WIRES

Since the measurements were conducted with Au bonding wires, the observed magnetization-independend thermopower is not only induced in the Py thin film but has also a contribution of the Au wires. This contribution can be estimated and excluded to find a ΔS purely for the Py thin film. As it was shown in the main text, the thermovoltage was extracted to be

$$U_{Seebeck} = -S_{+}\,|\nabla T|\,d = -(168 \pm 4)\,\mu V \quad . \tag{22}$$

Regarding the Seebeck coefficients of Py and Au ($S_{Py} = -4.5\,\frac{\mu\mathrm{V}}{\mathrm{K}}$ ref.[29], $S_{Au} = 1.8\,\frac{\mu\mathrm{V}}{\mathrm{K}}$ ref.[30]), the applied temperature difference between the electrical contacts can be estimated to

$$U_{Seebeck} = -S_{eff}\,\Delta T = -(S_{Py} - S_{Au})\,\Delta T \tag{23}$$

$$\Delta T = \frac{U_{Seebeck}}{-S_{eff}} = -(26.7 \pm 0.6)\,\mathrm{K} \quad . \tag{24}$$

ΔT fits very well to the applied temperature difference of 30 K and can be used to identify S_{-} with Eq. (8) in the main text

$$S_{-} = \frac{-U_{-}}{\Delta T} = \frac{-0.50\,\mu\mathrm{V}}{-26.7\mathrm{K}} = (0.019 \pm 0.002)\,\frac{\mu\mathrm{V}}{\mathrm{K}} \quad . \tag{25}$$

Now, ΔS can be derived only regarding the thermopower contribution of the investigated Py thin film

$$\Delta S = \frac{2S_{-}}{S_{Py} + S_{-}} = \frac{2\left(0.019\,\frac{\mu V}{\mathrm{K}}\right)}{\left(-4.5\,\frac{\mu V}{\mathrm{K}} + 0.019\,\frac{\mu V}{\mathrm{K}}\right)} = -(0.84 \pm 0.08)\% \tag{26}$$

[1] Thomson, W. On the electro-dynamic qualities of metals: effects of magnetization on the electric conductivity of nickel and of iron, Proc. Royal Soc. London **8**, 546 (1857)

[2] Thompson, D. A., Romankiw, L . T. & Mayadas, A. F. Thin Film Magnetoresistors in Memory, Storage, and Related Application, IEEE Trans. Mag. **11**, 1039 (1975)

[3] Ky, V. D. The Planar Nernst Effect in Permalloy Films, Phys. Status Solidi B **17**, K207 (1966).

[4] Meier, D. *et al.* Thermally driven spin and charge currents in thin $NiFe_2O_4$ /Pt films, Phys. Rev. B **87**, 054421 (2013).

[5] D. Meier *et al.* Longitudinal spin Seebeck effect contribution in transverse spin Seebeck effect experiments in Pt/YIG and Pt/NFO, Nat. Commun. **6**, 8211 (2015).

[6] Lang, W., Kühl, K. & Sandmaier, H. Absorbing layers for thermal infrared detectors, Sensors and Actuators A **34**, 243 (1992)

[7] Chen, J. & Erskine, J. L. Surface-step-induced magnetic anisotropy in thin epitaxial Fe films on W(001), Phys. Rev. Lett. **68**, (1992)

[8] Park, Y., Fullerton, E. E. & Bader, S. D. Growthinduced uniaxial inplane magnetic anisotropy for ultrathin Fe deposited on MgO(001) by obliqueincidence molecular beam epitaxy, Appl. Phys. Lett. 66, 2140 (1995), Appl. Phys. Lett. **66**, 2140 (1995)

[9] Kuschel, T. *et al.* Uniaxial magnetic anisotropy for thin Co films on glass studied by magnetooptic Kerr effect, J. Appl. Phys. **109**, 093907 (2011)

[10] Daboo, C. *et al.* Anisotropy and orientational dependence of magnetization reversal processes in epitaxial ferromagnetic thin films, Phys. Rev. B **51**, 15964 (1995)

[11] Zhan, Q., Vandezande, S. & van Haesendonck, C. Manipulation of in-plane uniaxial anisotropy in FeMgO(001)FeMgO(001) films by ion sputtering, Appl Phys. Lett. **91**, 122510 (2007)

[12] Zhan, Q., Vandezande, S., Temst, K. & van Haesendonck, C. Magnetic anisotropies of epitaxial Fe/MgO(001) films with varying thickness and grown under different conditions, New J. Phys. **11**, 063003 (2009)

[13] Kaibi, A. *et al.* Structure, microstructure and magnetic properties of $Ni_{75}Fe_{25}$ films elaborated by evaporation from nanostructured powder, Appl. Surf. Sci. **350** (2015)

[14] Li, X., Sun, X., Wang, J. & Liu, Q. Magnetic properties of permalloy films with different thicknesses deposited onto obliquely sputtered Cu underlayers, J. Magn. Magn. Mater. **377** (2015)

[15] Avery, A. D., Pufall, M. R. & Zink, B. L. Observation of the Planar Nernst Effect in Permalloy and Nickel Thin Films with In-Plane Thermal Gradients, Phys. Rev. Lett. **109**, 196602, (2012).
[16] Schmid, M. *et al.* Transverse Spin Seebeck Effect versus Anomalous and Planar Nernst Effects in Permalloy Thin Films, Phys. Rev. Lett. **111**, 187201 (2013).
[17] Meier, D. *et al.* Influence of heat flow directions on Nernst effects in Py/Pt bilayers, Phys. Rev. B **88**, 184425 (2013).
[18] Pu,Y., Johnston-Halperin, E. , Awschalom, D.D. & Shi, J. Anisotropic Thermopower and Planar Nernst Effect in $Ga_{1-x}Mn_xAs$ Ferromagnetic Semiconductors, Phys. Rev. Lett. **97**, 036601 (2006).
[19] Ky, V.D. Planar Hall and Nernst Effect in Ferromagnetic Metals, Phys. Status Solidi **22**, 729 (1967).
[20] Soldatov, I. V., Panarina, N., Hess, C. , Schultz, L. & Schäfer, R. Thermoelectric effects and magnetic anisotropy of $Ga_{1-x}Mn_xAs$ thin films, Phys. Rev. B **90**, 104423 (2014).
[21] Gurevich, A.G. & Melkov, G.A. Magnetization oscillations and waves, CRC Press, Inc. (1996).
[22] Kuschel, T. *et al.* Magnetic characterization of thin $Co_{50}Fe_{50}$ films by magnetooptic Kerr effect, J. Phys. D: Appl. Phys. **45**, 495002 (2012)
[23] Kuschel, T. *et al.* Magnetization reversal analysis of a thin B2-type ordered $Co_{50}Fe_{50}$ film by magnetooptic Kerr effect, J. Phys. D: Appl. Phys. **45**, 205001 (2012).
[24] Aharoni, A. Demagnetizing factors for rectangular ferromagnetic prisms, J. Appl. Phys. **83**, 3432 (1998).
[25] Yin, L.F. *et al.* Magnetocrystalline Anisotropy in Permalloy Revisited, Phys. Rev. Lett. **97**, 067203 (2006).
[26] Frait, Z., Kamberský, V., Ondris, M. & Málek, Z. On the effective magnetization and uniaxial anisotropy of permalloy films, Czech. J. Phys. B **13**, 279 (1963).
[27] Shestakov, A. S., Schmid, M., Meier, D., Kuschel, T. & Back, C. H. Dependence of transverse magnetothermoelectric effects on inhomogeneous magnetic fields, Phys. Rev. B **92**, 224425 (2015).
[28] Vansteenkiste, A. *et al.* The design and verification of MuMax3, AIP Advances **4**, 107133 (2014).
[29] Dejene, F. K. , Flipse, J. & van Wees, B. J. Spin-dependent Seebeck coefficients of $Ni_{80}Fe_{20}$ and in Co nanopillar spin valves, Phys. Rev. B **86**, 024436 (2012).
[30] Crisp, R. S. & Rungis, J. Thermoelectric Power and Thermal Conductivity in the Silver-Gold Allow System from 3-300K, Philosophical Magazine, **22**, 217 (1970).
